# Decoding the sorghum methylome: understanding epigenetic contributions to agronomic traits

**DOI:** 10.1042/BST20210908

**Published:** 2022-02-25

**Authors:** Ulduz Vafadarshamasbi, Emma Mace, David Jordan, Peter A. Crisp

**Affiliations:** 1 School of Agriculture and Food Sciences, The University of Queensland, Brisbane, QLD 4072, Australia; 2 Centre for Crop Science, Queensland Alliance for Agriculture and Food Innovation, Hermitage Research Facility, Warwick, QLD 4370, Australia; 3 Agri-Science Queensland, Department of Agriculture and Fisheries (DAF), Hermitage Research Facility, Warwick, QLD 4370, Australia

**Keywords:** crop improvement, epigenetics, epigenomics, methylation, sorghum, transposons

## Abstract

DNA methylation is a chromatin modification that plays an essential role in regulating gene expression and genome stability and it is typically associated with gene silencing and heterochromatin. Owing to its heritability, alterations in the patterns of DNA methylation have the potential to provide for epigenetic inheritance of traits. Contemporary epigenomic technologies provide information beyond sequence variation and could supply alternative sources of trait variation for improvement in crops such as sorghum. Yet, compared with other species such as maize and rice, the sorghum DNA methylome is far less well understood. The distribution of CG, CHG, and CHH methylation in the genome is different compared with other species. CG and CHG methylation levels peak around centromeric segments in the sorghum genome and are far more depleted in the gene dense chromosome arms. The genes regulating DNA methylation in sorghum are also yet to be functionally characterised; better understanding of their identity and functional analysis of DNA methylation machinery mutants in diverse genotypes will be important to better characterise the sorghum methylome. Here, we catalogue homologous genes encoding methylation regulatory enzymes in sorghum based on genes in *Arabidopsis*, maize, and rice. Discovering variation in the methylome may uncover epialleles that provide extra information to explain trait variation and has the potential to be applied in epigenome-wide association studies or genomic prediction. DNA methylation can also improve genome annotations and discover regulatory elements underlying traits. Thus, improving our knowledge of the sorghum methylome can enhance our understanding of the molecular basis of traits and may be useful to improve sorghum performance.

## Introduction

In higher plants, cytosine methylation at the 5′ carbon in genomic DNA (5mC) is a conserved chromatin modification that plays a pivotal role in the regulation of gene expression, transgene silencing, genomic stability, imprinting and inactivation of transposable elements (TEs) [1–4]. DNA methylation can be inherited through meiotic and mitotic divisions in plant cells [5] and is thus an epigenetic factor due to its heritable role in genome maintenance [6] and gene regulation [7,8]. DNA methylation states are mostly stably inherited in a mendelian fashion. However, methylation variants can cause variability in phenotypic behaviour due to spontaneous methylation changes that cause unexpected non-mendelian inheritance, as is the case in paramutation [9]. Variation in the patterns of DNA methylation may produce novel epialleles that may contribute to plant improvement and adaptation [10]. Profiles of DNA methylation can also be applied to refine genome annotations or identify putative *cis*-regulatory elements, as has been demonstrated in *Sorghum bicolor* (sorghum) [11,12].

Sorghum is an important cereal due to its adaptation to biotic and abiotic stress [13–15], and its usage in food, alcoholic beverages, animal feed and in biofuel production [16]. Its close relationship to the other major crops such as maize [17], sugarcane, and pearl millet [18] also makes sorghum an attractive focus for cereal genome research. Compared with *Arabidopsis* or even to other grasses like maize and rice, in sorghum there is more limited information about the regulation and natural variation for patterns of DNA methylation. In this review, we highlight what is known about the sorghum methylome, including similarities and differences to its close relative maize. Better elucidating methylation signatures in the sorghum genome may also be useful for sorghum improvement.

## Detection and distribution of DNA methylation in the sorghum genome

### Detection of DNA methylation

In plants, DNA methylation occurs in three sequence forms including CG, CHG and CHH, where H can be A, C, or T [19]. CG and non-CG contexts both have significant potential to be methylated [20]. Methods for DNA methylation detection include those based on bisulfite conversion, enzymatic digests that use combinations of methylation sensitive and insensitive restriction enzymes, and affinity-based immunoprecipitation methods [21–24]. Whole-genome bisulfite DNA sequencing or ‘WGBS’ is the gold-standard approach, which generates a base-pair resolution DNA methylation map [25]. WGBS based on chemical conversion can damage DNA and results in DNA degradation, so an enzymatic-based alternative was developed that uses 10–11 translation (TETs) dioxygenases to oxidise methylated cytosines (5-mCs), and convert unmethylated regions to uracils by catalytic polypeptide-like 3A (APOBEC3A) treatment [26]. Owing to the high cost of whole-genome profiling in large genomes, numerous cheaper alternatives have been developed, including sequence-capture approaches, array-based approaches and reduced-representation strategies analogous to genotype-by-sequence methods for genotyping [27,28]. However, these methods have significant limitations and progress in this field would be accelerated by the development of more economical profiling methods.

### Distribution of DNA methylation

**Figure 1.**
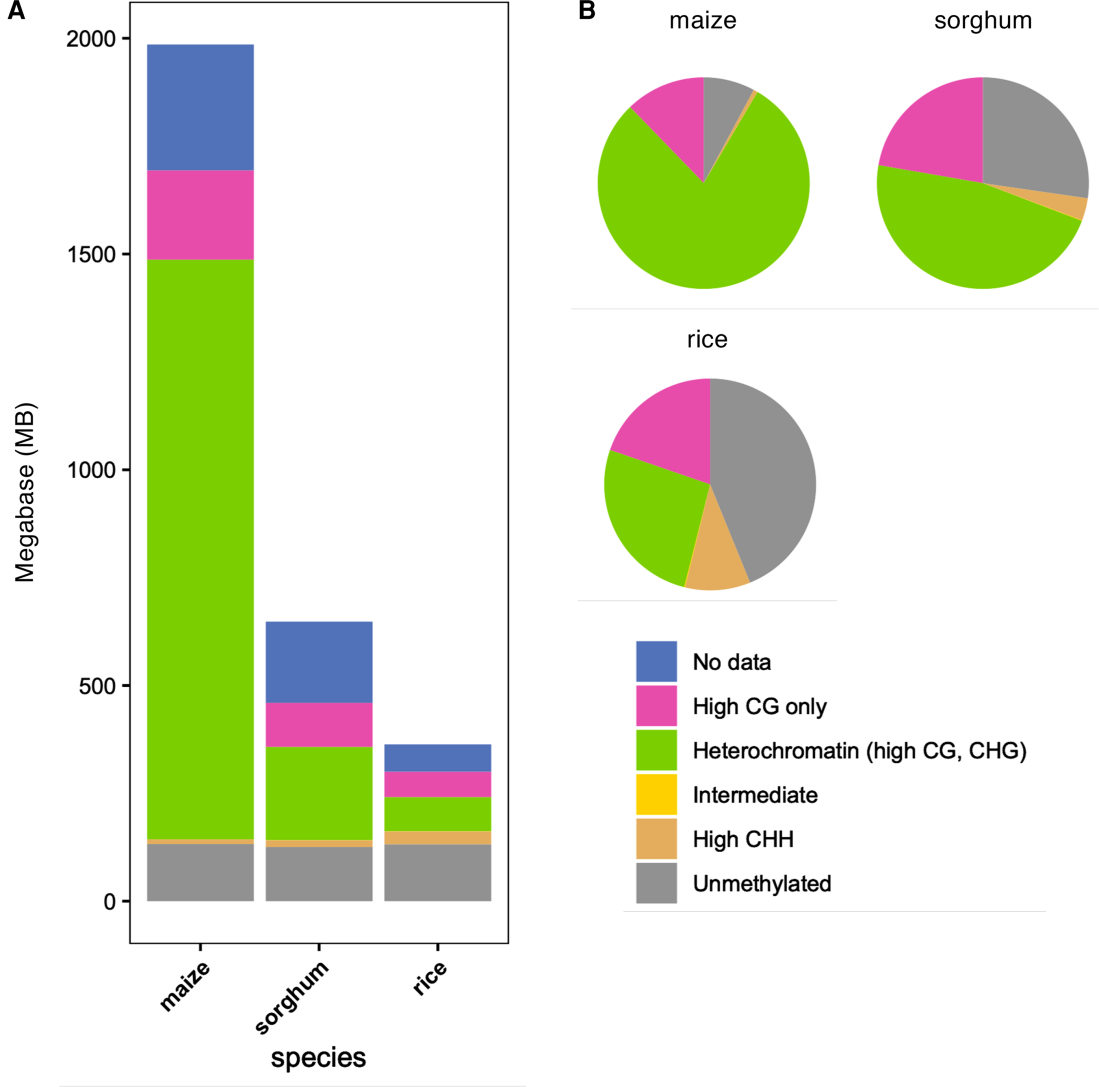
Comparison of DNA methylation domains between genomes. (**A**) The total base pairs of each methylation domain in the maize, sorghum, rice genomes. Data sourced from Crisp et al. [11]; (**B**) the relative abundance of different methylation domains in the maize, sorghum and rice genomes, expressed as a percent of each genome that can be analysed (excluding unmappable regions and regions with no data).

Plant species show extensive variation in both levels and patterns of 5′ cytosine methylation [29] (Figure 1A). Previous studies have profiled DNA methylation in sorghum [12,29–31], maize [6,32,33], rice [34] and many other species [35,36]. By profiling DNA methylation levels genome-wide, regions in the genome can be grouped into different domains based on the types and levels of DNA methylation, including regions with high CHH, CG, and CHG (‘High CHH'), regions with high CG and CHG only (‘Heterochromatin'), high CG only (‘CG-only' or gene body methylation), intermediate, and regions with low methylation in all contexts (‘Unmethylated'). For maize, sorghum, and rice the relative distribution of methylation domains vary; CG and CHG methylation occur in heterochromatic regions and are enriched at TEs and intergenic regions but depleted in genes and comprise 79% of the large genome of maize and 47% in sorghum, and only 26% in the smaller genome of rice (Figure 1A,B). These species vary in genome size, 389 Mb, 730 Mb, and >2 Gb in rice, sorghum, and maize, respectively (Figure 1A). Rice maintains ∼10% CHH methylation, the highest of these species, which is largely due to the RNA directed DNA methylation (RdDM) pathway [8]. The maize genome is methylated least at CHH sites with <1%. Rice has a higher rate of unmethylated regions at 43% compared with sorghum (27%) and maize (8%) (Figure 1B); however, in terms of absolute base pairs, the unmethylated space in each genome is remarkably similar, ∼125–132 Mb (Figure 1A).

**Figure 2.**
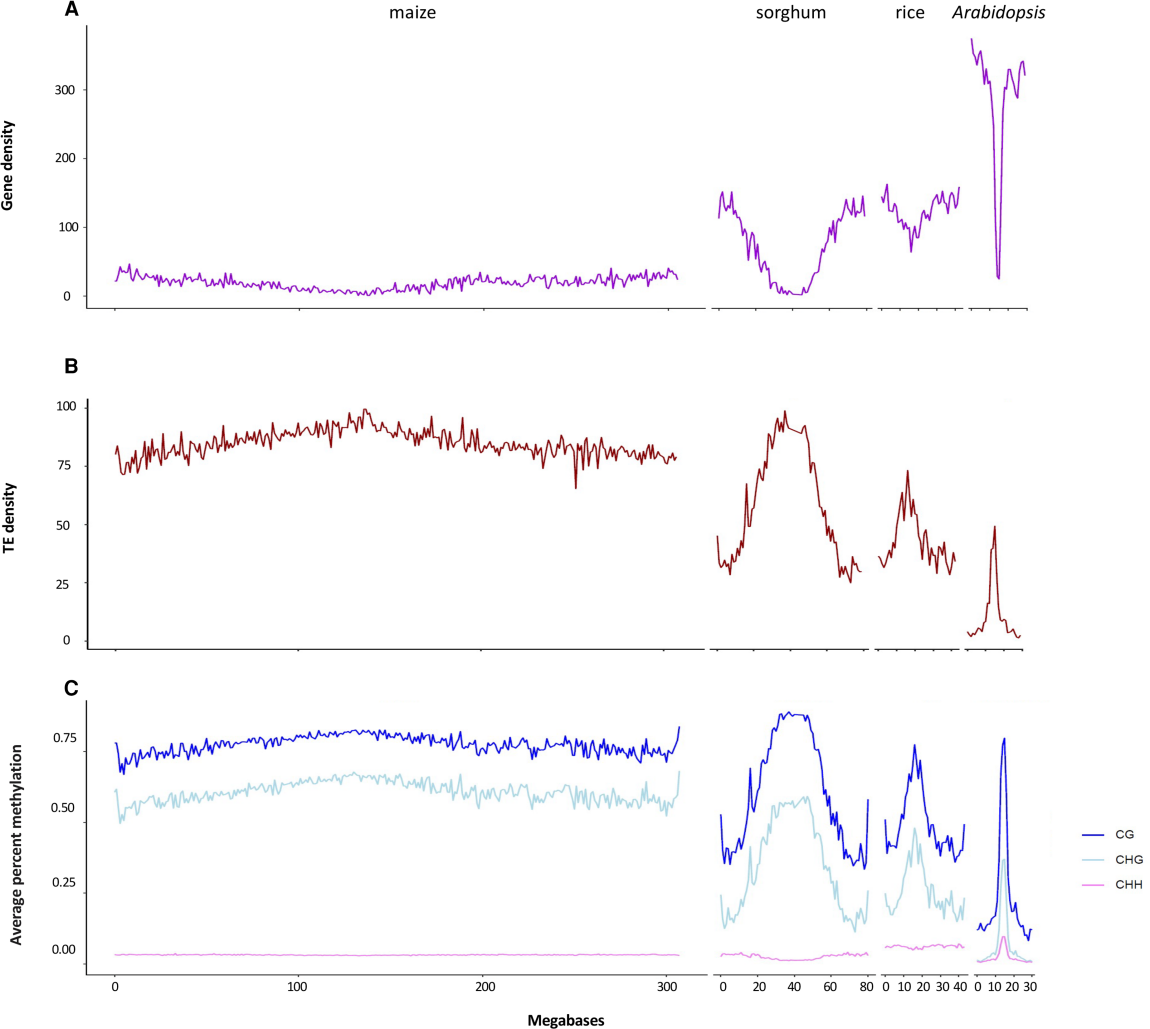
Distribution of genes, TEs and DNA methylation across maize, sorghum, rice, and *Arabidopsis* chromosome 1. (**A**) Gene density expressed as the total number of genes per megabase across chromosome 1 in each species; (**B**) distribution of TEs across chromosome 1 in each species expressed as percent of base pairs annotated as TE; (**C**) the average level of CG, CHG and CHH methylation per megabase. Methylation levels were determined per 100 bp tile and then averaged into megabase tiles across chromosomes for each species. Each chromosome is set to the same scale to illustrate the different size of maize (B73), sorghum (Btx623), rice (Nipponbare), and *Arabidopsis* (Col-0) chromosomes. Gene annotations sourced from Phytozome13, methylation data for maize, sorghum and rice sourced from Crisp et al. [11] and *Arabidopsis* from Crisp et al. [129]; TE annotations generated using EDTA [130] and the maize TE annotation generated using EDTA sourced from Hufford et al. [131].

Comparing sorghum to maize, rice and *Arabidopsis*, in each genome, gene density is highest in the chromosome arms and lower towards the pericentromeric regions, although relative gene density is much, much higher in the chromosome arms for sorghum compared with maize, rising to over 100 genes per megabase (Figure 2A), and higher again for Arabidopsis, with over 300 gene per megabase. For sorghum rice and *Arabidopsis*, the relative density of TEs and repeats displays an inverse pattern to gene density (Figure 2B). In contrast, TE density in maize is the highest of all species and is high throughout the chromosome. Likewise, the average DNA methylation levels along a chromosome varies between species (Figure 2C). For all, CG has the highest levels of methylation, followed by CHG, and CHH is the least methylated form. For all species, CHH has a low-level distribution pattern across the chromosome but is highest in rice, and also high in the pericentromeric regions of *Arabidopsis*. For maize, with its high abundance of TEs, CG and CHG are maintained at high levels right across the chromosome, while the proportion of CG- and CHG-methylated sites peaks around centromeric segments in sorghum, rice and *Arabidopsis* and are far more depleted in the gene dense chromosome arms (Figure 2C).

## Molecular mechanisms regulating DNA methylation

DNA methylation in plant genomes occurs with the addition of a methyl group (CH_3_) to cytosine residues, and is regulated by multiple complementary pathways and enzymes depending on the sequence context [1,37]. The components of the methylation pathways have been extensively studied in the model plant *Arabidopsis* [38,39] and also in maize [40] and rice [41,42]. In *Arabidopsis*, these components have been knocked out individually and in combination to reveal many important insights into methylation in each sequence context [39]. However, in larger crop genomes, mutants in many components have not been obtained despite attempts, suggesting these genes are indispensable [40], which has hampered functional analysis. In rice, CRISPR/Cas disruption of each methyltransferases individually led to viable plants except for MET1 loss of function [42]. Most homologous genes encoding methylation regulatory enzymes in maize have been identified [43]. Here, we review the sorghum gene homologues based on genes identified in *Arabidopsis*, maize, and rice; however, functional studies in sorghum are an important area for future research.

### Initiating and maintaining methylation

Cytosine-5′ DNA methyltransferases (C5′-MTases) catalyse the addition of methyl groups with a complementary role for ‘*de novo*’ and ‘maintenance’ in regulating methylation [29]. C5′-MTases consist of three main enzyme groups: (1) DOMAINS REARRANGED METHYLTRANSFERASES (DRMs) that catalyse *de novo* methylation in all three methylation contexts through RNA-directed DNA methylation (RdDM) [44]; (2) METHYLTRANSFERASES (METs) that are responsible for the maintenance of CG methylation at each round of DNA replication [45]; and (3) CHROMOMETHYLASES (CMTs) that catalyse the maintenance of CHG and CHH methylation [46,47].

*De novo* DNA methylation is guided by small interfering RNAs (siRNAs) and scaffold RNAs via the RdDM pathway [19,38]. The siRNA precursors and scaffold RNAs are transcribed by two plant specific RNA polymerase II (Pol II)-related enzymes including Pol IV and Pol V [48,49]. In the ‘canonical’ RdDM pathway, Pol IV produces a single-stranded RNA (ssRNA). The ssRNA is reverse-transcribed by RNA-DEPENDENT RNA POLYMERASE 2 (RDR2) to produce a double stranded RNA (dsRNA), which is finally cleaved to 24 nucleotide (nt) siRNAs [41]. During canonical RdDM, Pol IV and Pol V are recruited to target loci by other heterochromatic marks and co-factors, for example SAWADEE HOMEODOMAIN HOMOLOG 1 (SHH1) that binds histone H3 lysine 9 methylation (H3K9me2) [50,51]. The initiation of RdDM has been more enigmatic; however, recent findings suggest that the self-reenforcing cycle of RdDM is initiated by an AGO4 clade ARGONAUTE guided by an siRNA to a locus independent of pre-existing DNA methylation [52].

In plants, the RdDM pathway recruits DRMs for maintenance of CHH methylation [38,53]. In rice, OsDRM2 plays a major role in CHH methylation [8]. The siRNAs binding to ARGONAUTE 4 (AGO4) or AGO6 and scaffold RNAs activate the enzyme DOMAINS REARRANGED METHYLTRANSFERASES 2 (DRM2) for DNA methylation at the target loci [53,54]. CMT2 in *Arabidopsis* also plays a role in the maintenance of CHH methylation [46]; while maize lacks a CMT2 homologue, sorghum does have a homologue, SbCMT2 (Sobic.009G083900). In the maize genome, DRM-like genes ZMET3 and ZMET7 are homologous to *Arabidopsis* DRM1 and DRM2, and ZMET6 that is related to DRM3 [40]. Homologues of DRM1/DRM2, and DRM3 in sorghum are SbDRM2 (Sobic.003G124000) and SbDRM3 (Sobic.009G032200) (Table 1).

**Table 1:** Methylation and Demethylation genes in sorghum

Gene		Sorghum gene	Sorghum identifier	Homologues		
				Maize	Rice	Arabidopsis [75]
Methylation	MET1b	SbMET1b	Sobic.002G056000	ZmMET1b-1 (Zm00001eb301630) and ZmMET1b-2 (Zm00001eb301620) [43]	OsMET1-2 (LOC_Os07g08500) [55]	AtMET1 (AT5G49160)
	MET1a	SbMET1a	Sobic.001G055700		OsMET1-1 (LOC_Os03g58400) [55]	AtMET1 (AT5G49160)
	CMT3/Zmet	SbCMT3a	Sobic.004G197400	ZMET5 (Zm00001d002330 v4 *missing in maize v5) [40]	OsCMT3a (LOC_Os10 g01570) [41,62]	AtCMT3 (AT1G69770)
	CMT3/Zmet	SbCMT3b	Sobic.006G214000	ZMET5 (Zm00001d002330) and ZMET2 (Zm00001d026291) [40]	OsCMT3b (LOC_Os03g12570) [41]	AtCMT3 (AT1G69770)
	DDM1a	SbDDM1a	Sobic.002G021200	CHR101 (ZmDDM1A; Zm00001eb117870)	OsDDM1a (LOC_Os09g27060) [8]	AtDDM1 (AT5G66750)
	DDM1b	SbDDM1b	Sobic.001G109700	CHR101 ZmDDM1A; Zm00001eb117870) or CHR106 (ZmDDM1B; Zm00001eb055640) [40,64]	OsDDM1b (LOC_Os03g51230) [8]	AtDDM1 (AT5G66750)
	CMT2	SbCMT2	Sobic.009G083900		OsCMT2 (LOC_Os05g13780) [46]	AtCMT2 (AT4G19020)
	DRM1/DRM2	SbDRM2	Sobic.003G124000	ZMET3 (Dmt103; Zm00001eb404130), ZMET7 (Dmt107; Zm00001eb000820) [40]	OsDRM1a (LOC_Os11g01810); OsDRM1b (LOC_Os12g01800); OsDRM2 (LOC_Os03g02010) [41]	AtDRM1 (AT5G15380)
	DRM3	SbDRM3	Sobic.009G032200	ZMET6 (Dmt106; Zm00001eb354730) [40]	OsDRM3 (LOC_Os05g04330) [42,76]	AtDRM1 (AT5G15380)
Demethylation	ROS1	SbROS1c	Sobic.008G085300	DNG102 (Zm00001eb241240) [43,74]	OsROS1a (LOC_Os01g11900) OsROS1b-d (LOC_Os05g37410, LOC_Os05g37350, LOC_Os02g29230) [73,77]	AtROS1 (AT2G36490); AtDML2 (AT3G10010)
	ROS1	SbROS1b	Sobic.004G149800	DNG101 (Zm00001eb202980) [43,74]	As above	As above
	ROS1	SbROS1a	Sobic.009G155900	DNG103 (Zm00001eb289030) [43,74]	As above	As above
	DML3	SbDML3	Sobic.006G224100	DNG105 ZmDML3 (Zm00001eb241310) [73]	OsDML3a-b (LOC_Os04g28860; LOC_Os02g29380) [41]	AtDML3 (AT4G34060)

To identify putative genes in sorghum associated with methylation and demethylation, the homologous genes previously annotated for Arabidopsis, maize and rice genomes were identified through literature searches (as cited) and each pairwise aligned to the sorghum genome using the BLAST tool (NCBI).

Following DNA replication, the methylation patterns for CG, CHG, and CHH are maintained by different mechanisms [38]. DNA METHYLTRANSFERASE 1 (MET1) is responsible for maintenance of CG sites in *Arabidopsis* [38]. The rice genome encodes two redundant MET1 genes, OsMET1-1 and OsMET1-2 that maintain methylation at CG sites, with higher levels of OsMET1-2 expression [55]. The maize genome encodes two tandemly duplicated MET1-like genes that are both in the monocot MET1b clade [56], including ZmMET1b-1 (Zm00001eb301630) and ZmMET1b-2 (Zm00001eb301620) [43]. In sorghum, there is a homologue of rice OsMET1a, SbMET1a (Sobic.001G055700) and a homologue of the maize ZmMET1b duplicates, SbMET1b (Sobic.002G056000) (Table 1).

The methylation of CHG sites is maintained by chromomethylases (CMTs) in a reinforcing loop and requires the activity of a chromodomain, which recognises specific histone modifications [41]. In this loop, histone H3 lysine 9 di-methylation (H3K9me2) is catalysed by the H3K9 methyltransferases KRYPTONITE (KYP)/SUPPRESSOR OF VARIEGATION 3–9 HOMOLOGUE 4/5/6 (SUVH4/5/6) at genomic regions [57,58]. *Arabidopsis* has three CMT genes: CMT1, CMT2 and CMT3 [4], although CHG methylation relies on the chromomethylases CMT3 rather than CMT2 [47,57] and CMT1 function is unknown. There are in-paralogs of CMT3 in maize ZMET2 and ZMET5 that catalyse the maintenance of CHG sites [40,59], that have a close relationship with CMTs in other monocots but the maize genes are considered a separate subclade and named ZMETs . Based on the genealogical relationship between CMTs and ZMETs, it is suggested that they have been derived from an ancestral whole-genome duplication among angiosperms [60]. Maize ZMET2 seems to contribute to the maintenance of CHG as a main functional homologue with CMT3, while its paralog ZMET5 is expressed to lesser degree [40,59]. ZMETs in sorghum also arose from two independent duplications, which might have been derived from Poaceae-specific duplication events [61]. However, there are differences between genes and amino acids of ZMETs in sorghum compared with maize [61]; the sorghum homologues are SbCMT3a (Sobic.004G197400) and SbCMT3b (Sobic.006G214000) (Table 1). In rice, three CMT genes including OsCMT2, OsCMT3a, and OsCMT3b have been identified; however OsCMT3a takes charge of maintaining CHG sites as the main functional homologue of CMT3 that contributes in the expression of genes and TEs, while OsCMT3b seems to contribute at minor level [42,62]. The chromomethylase CMT2 recognises H3K9me2 within deep heterochromatin enriched with TEs to methylate CHH [63]. Rice, unlike maize, does have a functional CMT2 homologue, OsCMT2 [62], which is also homologous to sorghum SbCMT2 (Sobic.009G083900) (Table 1).

The maintenance of DNA methylation is also regulated by chromatin remodellers [41]. DECREASE IN DNA METHYLATION1 (DDM1) is a nucleosome remodeller, and is required to maintain DNA methylation in *Arabidopsis* especially at TEs [46]. The two homologues of DDM1 in maize (ZmDDM1a and ZmDDM1b), are highly expressed with similar patterns including in the embryo [40,64] and play a critical role for CHG methylation, and to a lesser extent for CGs in heterochromatic regions. DDM1 also contributes to methylation of CHH islands, located in euchromatic regions in interaction with ZmAGO4 [65,66]. In sorghum, DDM homologues are SbDDM1a (Sobic.002G021200) and SbDDM1b (Sobic.001G109700) (Table 1).

### Demethylation

In addition to *de novo* methylation and maintenance, demethylation can play a critical role in plants, for example, controlling fruit ripening, in gametophytes, and in plant aging and senescence [67,68]. In plants, demethylation events occur through the base excision repair pathway, which itself is an essential defense pathway for the genome and performs the replacement of 5-methylcytosine with cytosine in an active demethylation process [69]. Passive demethylation also contributes to demethylation, where 5-methylcytosine is diluted from the genome during DNA replication [4]. In *Arabidopsis*, four genes have been identified as 5-methylcytosine DNA glycosylases (DNGs) belonging to three distinct orthology groups, the REPRESSOR OF SILENCING 1 (ROS1), DEMETER (DME) and two DEMETER-Likes, DML2 and DML3 [70]. ROS1 plays an essential role in deactivation of methylated cytosines and inhibits transcriptional silencing of both transgenic and endogenes loci in the *Arabidopsis* genome [71]. It is also reported that there is a dynamic counteraction between the RdDM and DNA demethylation for ROS1 expression, so that RdDM targets methylation in the promoter of ROS1, but in another way, prevents ROS1 hypermethylation by inducing ROS1 transcription that leads to active DNA demethylation [71,72]. Monocots lack DME orthologs but do have ROS1 and DML orthologs [73]. Two homologues in the maize genome for ROS1; DNG101 and DNG103 have been reported [43]. Sorghum genes of the DNG family include SbROS1a, SbROS1b, and SbROS1c that are homologous to DNG103 (Zm00001eb289030), DNG101 (Zm00001eb202980), and DNG102 (Zm00001eb241240) in maize [43,74] (Table 1). The sorghum genome also encodes a homologue to ZmDML3 [73], SbDML3 (Sobic.006G224100), that is a DME-like gene, (Table 1). However, the functional activity of these sorghum demethylation pathway genes needs to be experimentally explored.

## Opportunities and challenges for crop improvement

Variation in DNA can underpin phenotypic traits of agronomic relevance [7,78,79]. In this section, we highlight areas that require a better understanding in sorghum in order to leverage the methylome for crop improvement, and discuss avenues for implementation. It is not yet clear if some traits might be more amenable to improvement via epigenetic approaches; however, it will be valuable to investigate this for key targets for agronomic improvement. Example key traits include, yield and yield components such as grain size and number; abiotic stress tolerance, including cold tolerance at emergence, heat tolerance during flowering and drought tolerance; and grain quality and digestibility.

### Heritability

Patterns of symmetric DNA methylation can be faithfully maintained and inherited through cell divisions and even CHH methylation can be propagated via the RdDM pathway. This means that patterns of methylation are highly stable for much of the life cycle of plants, especially during vegetative development. That said, tissue specific differences in methylation are reported, including in sorghum [30,80]. Epigenetic reprogramming causes changes in DNA methylation states during gametogenesis [81], and in turn affects reproductive lineages. Notably, the agronomically valuable cereal endosperm is one tissue where DNA methylation levels are reduced compared with other tissues, which has been observed in rice [82], maize [83] and sorghum [80].

The heritability of DNA methylation states can be defined by the three classes of epiallele: obligatory, facilitated, and pure [84]. Obligatory epialleles are a direct result of *cis*- and *trans*-acting sequence variation. As an example, an insertion of a transposon can be associated with epigenetic silencing of neighbouring coding sequences. Facilitated epialleles are also linked to genetic variation but can act independently; an example is where a transposon causes methylation in a nearby gene but then is segregated out [5,84]. Facilitated epigenetic variation could occur due to either local or cis-acting genetic variants or trans-acting variants [85,86]. Lastly, pure epialleles are entirely independent of genetic variation, and are generated by the activity (or failure) of the DNA methylation/demethylation pathways [84]. Changes in methylation for epialleles have a potential to persist and pass onto the next generations as epigenetic alleles independent of the underlying DNA sequence, and could influence phenotypic variation in traits making them of interest to applied breeding programs [87]. Importantly, only obligatory and cis-acting facilitated epialleles might be already captured by genetic marker-based (e.g. SNP) selections schemes, while epialleles generated by trans-acting variants and pure epialleles are likely to escape genetic marker-based selection [86].

For a particular genomic region, methylation levels can be categorised as non-methylated, low, partial, or high-methylated, and the heritability of these states range from high stability to entire instability [86,88]. Epigenetic variation derived from trans-acting genetic factors and genetic variants may cause a paramutation, which is an interaction between alleles for a specific region/locus that potentially alter mendelian inheritance and lead to a heritably altered gene expression state through mitosis [89]. Epigenetic alleles or ‘epialleles’ can drive changes in phenotype across generations in the absence of any DNA sequence changes (SNPs) [90]. Spontaneous or random errors in the maintenance of methylation lead to ‘epimutation’ and generate single methylation polymorphisms (SMPs) including CG-, CHG-, CHH-SMPs [91], or differentially methylated regions (DMRs) [92]. Spontaneous epimutation rates are estimated to be similar across species [93], with SMPs in the range 1 × 10^−4^ to 10^−5^ sites/haploid/generation, which is far higher than SNPs (1 × 10^−8^ to 10^−9^) [86]. In contrast with SMPs, the level of methylation across larger regions is more stable (1 × 10^−5^ to 10^−6^ sites/haploid/generation) [94,95]. Even in the face of environmental stress, DNA methylation is highly stable over generations [94]. DMRs among genotypes are very heritable, although they have lower stability in comparison with SNPs [86,92], which can be due to spontaneous backward epimutation in CG sites [96]**.**

### Natural variation

Comparisons of DNA methylation from different varieties or genotypes within a species have identified extensive natural variation. However, for most species and especially for crop species, profiling large populations is very limited. Extensive variation has been identified in *Arabidopsis* [91,97], the only species to have been profiled on a population scale. Consistent with findings in *Arabidopsis*, smaller-scale studies in maize, soybean, rice, wheat and brachypodium [86] point to extensive variation, including variants associated with domestication. Likewise, sequence capture profiling of ∼0.2% of the hexaploid wheat genome [98] identified diversity associated with agronomic traits among 104 varieties [99]. Profiling of 263 diverse maize varieties found many epialleles that were associated with gene expression changes and metabolic traits [6]. Importantly, around half of the trait associated regions with methylation polymorphisms identified in this study would not have been identified by profiling SNPs. The extent and spectrum of natural variation within sorghum is an exciting area for future research.

### Inducing variation

Future efforts that seek to use epialleles for sorghum improvement could take advantage of several important sources of induced variation including chemical treatments, tissue culturing and genetic and epigenetic mutation. For example, new epialleles for the gene *pericarp color1* (*p1*) derived from tissue culture in maize, showed loss of pigmentation [100]. Also, induced epigenetic changes during tissue culture in rice were reported to be inherited in regenerated plants [101], and can also lead to activation of the Tos17 retrotransposon [102–105]. Tissue culture induced changes in DNA methylation have also been observed in sorghum using MSAP profiling [106]. Mutation in MET1 or DDM1 globally decreases CG methylation [7], which can be used to generate epigenetic recombinant inbred lines (epiRILs). epiRILs have been very useful in investigating the epigenetic basis of traits, although populations in large genome crop species are yet to be generated [5]. Chemicals such as zebularine can also be used to generate populations of epi-mutagenised plants, which has been applied to wheat to select for variation in flowering time and spike morphology [107]. Going forward, the use of epi-genome editing will be a powerful alternative means to precisely generate or investigate epialleles [2]. Some studies have also found that the environment can induce changes in methylation. For example, a study on root system architecture found methylation variation associated with environmental stresses [108]; other studies have found that DNA methylome is stable under stresses [94].

### Use of epigenetic variation in trait dissection

The most straightforward application of DNA methylation in breeding would be to use methylation marks for selection of epi-haplotypes associated with phenotypes of interest [109]. The impact of such an approach would require that epialleles were frequent and explained significant amounts of phenotypic variation and were stably transmitted [110,111]. Data from a range of crops suggests this may indeed be the case. Epigenetic variants have been shown to contribute to agronomic traits such as dwarf phenotypes in rice [112,113], drought and salt tolerance in rice [114], increase in protein and decrease in oil contents in *Brassica napus* [115]. *NMR19-4* was reported as a stable environmental-associated epiallele that negatively regulates the expression of a PHEOPHYTIN PHEOPHORBIDE HYDROLASE (PPH), which may result in variations in leaf senescence [116]. Thus, epigenome-wide association studies (EWAS) could complement genome association studies [117,118]. Several DMRs in *Arabidopsis* are epiQTLs that control flowering time and root length, and accounting for between 60–90% stable inheritance, which could be used as epi-markers for selection trials [119]. EWAS has been used in maize to identify genomic regions with differential methylation associated with traits that would be missed by SNP based GWAS alone [6].

Genetic mapping and GWAS studies play a crucial role in our ability to understand the genetic architecture of complex traits and identify markers for major genes that can be used in marker-assisted selection. Yet, sometimes these markers fall outside of genes. Chromatin profiling and the patterns of DNA methylation can be used to refine genome annotations and identify functional genomic elements [120]. In sorghum, DNA methylation profiles have been used to identify high confidence gene models from draft annotations [121]. Identifying the unmethylated portion of the genome is also a powerful method to identify putative *cis*-regulatory elements and expressed genes and has been successfully applied to sorghum [11]. This use of methylation in this way can aid in better understanding of the sorghum genome and the molecular basis of traits including quantitative trait loci (QTLs), which can contribute to crop improvement molecular breeding strategies.

### Use of epigenetic variation to enhance genomic selection

DNA methylation signatures also have potential to be incorporated in genomic selection schemes [122]. For example, in *Arabidopsis* DNA methylation data has been used to predict plant height [123]. Inclusion of epialleles in genomic selection models would require research to identify the best way to exploit this data and integrate it into genomic selection models, however, it will be very interesting to see if this can improve predictive power. In addition, prediction for performance of F1 hybrids remains challenging; genetic information is not sufficient for predicting heterosis perfectly, epigenome profiles may improve genome prediction due to epigenetic contributions to heterosis [124,125]. Thus, epigenetic modelling has the potential to fill current gaps and enhance our ability to predict phenotypic behaviour [126].

### Use of epigenetic variation to modify meiotic recombination

Meiotic recombination has a critical influence on genetic gain in plant breeding programs. In some situations, high recombination can be favourable, such as when it is necessary to reduce the impact of linkage drag while in other situations lower recombination may be helpful in retaining favourable linkage blocks. Changes in methylation in both CG [127] and non-CG [128] contexts in pericentromeric heterochromatin in *Arabidopsis* has been shown to increase meiotic recombination. It is conceivable that if methylation can be manipulated in sorghum, that could be used to favourably change recombination rate to enhance progress in breeding programs, for example by enabling access to variation in regions of low recombination.

## Perspectives

- DNA methylation is a chromatin modification that may provide an untapped layer of information on top of DNA sequence.
- This information is heritable, therefore, DNA methylation may provide a mechanism of epigenetic inheritance of trait variation that could be helpful in sorghum crop improvement. The sorghum DNA methylome and the genes regulating DNA methylation are understudied.
- It will be critical to determine the extent of variation for DNA methylation and the heritability of these epialleles in sorghum because this information could be very useful in selection, genomic prediction, pre-breeding and research, and complement current genetic approaches.

## Abbreviations

AGO4: ARGONAUTE 4
CMTs: chromomethylases
DDM1: DECREASE IN DNA METHYLATION1
DME: DEMETER
DMRs: differentially methylated regions
DRM2: DOMAINS REARRANGED METHYLTRANSFERASES 2
EWAS: epigenome-wide association studies
MET1: METHYLTRANSFERASE 1
SMPs: single methylation polymorphisms
TEs: transposable elements
